# Elucidating the novel biomarker and therapeutic potentials of High-mobility group box 1 in Subarachnoid hemorrhage: A review

**DOI:** 10.3934/Neuroscience.2019.4.316

**Published:** 2019-12-02

**Authors:** Seidu A. Richard

**Affiliations:** Department of Medicine, Princefield University, P. O. Box MA 128, Ho-Volta Region, Ghana West Africa

**Keywords:** CSF, HMGB1, Plasma, Prognosis, SAH, Vasospasm

## Abstract

Subarachnoid hemorrhage (SAH) frequently arises after an aneurysm in a cerebral artery ruptures, resulting into bleeding as well as clot formation. High-mobility group box 1 (HMGB1) is an extremely preserved, universal protein secreted in the nuclei of all cell varieties. This review explores the biomarker as well as therapeutic potentials of HMBG1 in SAH especially during the occurrence of cerebral vasospasms. Plasma HMGB1 levels have proven to be very useful prognosticators of effective outcome as well as death after SAH. Correspondingly, higher HMGB1 levels in the cerebrospinal fluid (CSF) of SAH patients correlated well with poor outcome; signifying that, CSF level of HMGB1 is a novel predictor of outcome following SAH. Nonetheless, the degree of angiographic vasospasm does not always correlate with the degree of neurological deficits in SAH patients. HMGB1 stimulated cerebral vasospasm, augmented gene as well as protein secretory levels of receptor for advance glycation end product (RAGE) in neurons following SAH; which means that, silencing HMGB1 during SAH could be of therapeutic value. Compounds like resveratrol, glycyrrhizin, rhinacanthin, purpurogallin, 4′-O-β-D-Glucosyl-5-O-Methylvisamminol (4OGOMV) as well as receptor-interacting serine/threonine-protein kinase 3 (RIPK3) gene are capable of interacting with HMGB1 resulting in therapeutic benefits following SAH.

## 1. Introduction

Subarachnoid hemorrhage (SAH) frequently arises after an aneurysm in a cerebral artery ruptures, resulting into bleeding as well as clot formation [1]. In some cases, the parent artery ruptures spontaneously without an aneurysm [1],[2]. SAH accounts for about 5–10% of all strokes. SAH is a disorder with very unique as well as life threatening clinical challenge [3]. Death occurs in about 30–50% of patients who suffer SAH while 40–50% of patients who recover from the event experience major neurological deficits [1],[4]. The quantity of blood as well as the size of the clot formed often predicts the sternness of the event as well as its outcome [1],[5]. Hypertension, old age, alcohol misuse as well as cigarette smoking have been implicated as foremost risk factors associated with SAH [1],[6]. Cerebral vasospasm is the most serious complication after the occurrence of SAH [5],[7].

Nearly 70% of patients who suffer SAH seem to be recovering well during the first 1–2 days after the cerebral event, but as the days go by, cerebral vasospasm complicates this recovery process [1],[4]. Currently, the most accurate diagnostic and monitoring modality for SAH is radiology. Although several chemical biomarkers have been implicated as predicting monitoring biomarkers during SAH, high-mobility group box 1 (HMGB1) is the most promising. This review therefore explores the biomarker as well as therapeutic potentials of HMBG1 in SAH especially during the occurrence of cerebral vasospasms.

## 2. HMGB1

HMGB1 is present in the nuclei and it is secreted from nuclei into cytoplasm and then extracellularly upon injury [8]–[10]. It is one of the archetypes of the supposed alarmin family [11]–[13]. It has been implicated in DNA bending, sustaining nucleosome configuration as well as modulating gene transcription [14]. Studies have affirmed that, HMGB1 is expressed by necrotic cells or actively expressed by immune cells as well as non-immune parenchymal cells in several diseases [15]–[17]. Studies have shown that, during aneurysm rupture, HMGB1 remarkably partakes in sterile inflammation [11],[16],[18],[19]. It is clearly affirmed that, HMGB1 is secreted by every part of the nucleus in normal brain cells especially cells like neurons, astrocytes, and microglia [11],[20]. Also, HMGB1 has demonstrated to be subversive in immunological cells such as macrophages and monocytes [11],[20],[21]. It is proven that, HMGB1 facilitates inter-communication between damaged cells as well as comparatively healthy cells around injured tissues [22],[23]. Current research has indicated that HMGB1 is a potential biomarker for the interpretation of neurologic sequel in SAH patients [20].

Wang et al established that HMGB1 secretion was up-modulated in the cortex after SAH [24]. They utilized double immunofluorescence staining to detect that most cells that were positive for HMGB1 were also positive for NeuN/NSE [24]. This signifies that, HMGB1 secretion by the neurons were the primary source of elevated HMGB1 by the cortex after SAH [24]. It is established that, HMGB1 intermediate in vascular monocyte chemotaxis, neuron dendrite outgrowth, as well as proinflammatory response in endothelial cells during SAH [25]–[27]. Furthermore, HMGB1 is able to initiate inflammation as well as tissue repair. It also has the potentials of recruiting inflammatory cells, enticing stem cells as well as stimulating their proliferation. The reactions above often result in expression of monocytes, macrophages, neutrophils, platelets as well as microglia during SAH [25],[28],[29].

The stimulation of monocytes, macrophages, circulating neutrophils as well as platelets result in delayed expression of HMGB1 [23],[27],[30]. Sun, et al detected the expression of HMGB1 from the neurons 2 hours after SAH [23]. They observed elevation in inflammatory factors like TLR-4, NF-κB, IL-1β, as well as cleaved Caspase-3 after intraventricular injection of recombinant HMGB1 (rHMGB1) [23]. Also, introduction of hemoglobin (Hb) during an in-vitro study resulted in the elevation as well as translocation of HMGB1 from nucleus to cytoplasm in neuronal cultures [31].

## 3. Dual secretion of HMGB1 during SAH

Studies have proven that, the expression of HMGB1 from injured cells is both passive as well as active via translocation from the nucleus to the cytoplasm [23],[32]. It has been established that, cytosolic HMGB1 are higher in brain parenchyma of SAH animals as compare to normal controls [32],[33]. It has also been established that, both passive and active expression of HMGB1 are associated with cytokine actions resulting in inflammatory reactions [34]. In neurons, HMGB1 was detected to translocated from the nucleus to the cytoplasm 2 hours after SAH [23]. Subsequent to translocation, HMGB1 as well as its mRNA levels were markedly elevated [23]. It was affirmed that, both passive as well as active secretion of HMGB1 were intricate in the translocation process of HMGB1 in neurons during SAH [23]. Also, it has been established that, the secretion of rHMGB1 or HMGB1 from neurons triggered inflammatory reactions and extracellular HMGB1 participated in immediate brain injury after SAH [23].

It is further confirmed that, HMGB1 is expressed from blood clots near the cortex 2 hours after SAH [23]. This fact was established with western blot as well as immunhistochemistry analysis. Friedrich et al observed that, cortex cell death developed 10 minutes after SAH [35]. Thus, passive expression of HMGB1 was triggered by injured cellular integrity while active release of HMGB1 was likewise sustained by up-modulated mRNA as well as protein levels of HMGB1 [23]. Nevertheless, the translocation of HMGB1 often heralded the upsurge of more cytokines. This means that HMGB1 partakes in early up-regulation of inflammation after SAH. Therefore, early-expression of HMGB1 from neurons is an early up-regulatory determiner during inflammatory reaction after SAH [23].

Also, HMGB1 expression from neurons stimulates neighboring glial cells as well as up-modulation of inflammatory factors which results in the activation of brain cells to secret extra HMGB1 leading to up-modulation of mRNA level of HMGB1 [16],[23]. Furthermore, it has been detected that, minute quantity of microglia secrets HMGB1 at the early phase of SAH [23]. Nevertheless, it is presumed that, several microglia begin to express HMGB1 with time, usually at the late phase of SAH [33]. This occurrence was also observed at late phase ischemic brain injury [36]. Therefore, HMGB1 expression from microglia also contributes significantly to inflammation during the late phase of SAH [23].

## 4. HMGB1 levels in serum during SAH

**Figure 1.**
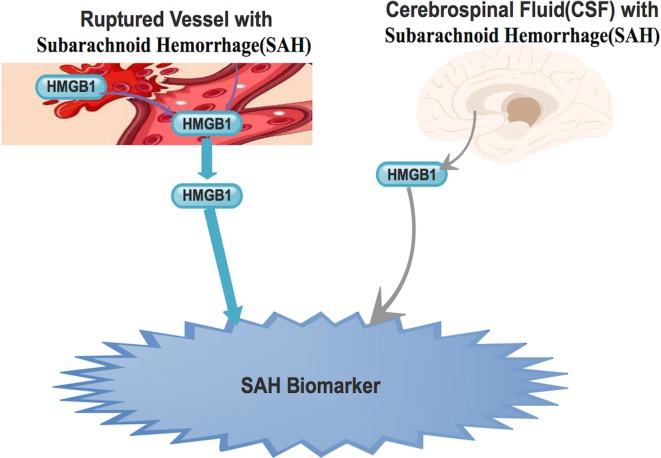
Ruptured vessel as well as the endothelium are able to secreted HMGB1 into the blood stream. Also, during SAH, HMGB1 is expressed into the CSF. The expressive levels in both plasma and CSF are high enough and thus could be used as biomarker during SAH.

Studies have demonstrated that, plasma HMGB1 levels are very useful prognosticators of efficient outcome as well as death after SAH [20],[32] Figure 1. It is affirmed that, admission plasma HMGB1 levels were certainly expressively elevated in all patients with SAH compared with healthy controls [32]. It is further established that, in-patient plasma HMGB1 levels are as consistent as well as autonomous marker to envisage patient's mortality, cerebral vasospasm as well as prognosis. Also, the prognostic levels of HMGB1 were analogous to those of world federation of neurosurgical societies (WFNS) score as well as modified Fisher score for in-patient mortality as well as prognosis [32]. It is affirmed that one-year prognostic results indicate that, HMGB1 is potential prognostic biomarker [20],[32].

Haruma et al revealed that, the injection of anti-HMGB1 mAb to rats with established SAH blocked the translocation as well as expression of HMGB1 in smooth muscle cells (SMCs) [37]. Also, they further observed diminished HMGB1 levels in the basilar artery. Nevertheless, the simultaneous measurement of plasma HMGB1 also showed that the anti-HMGB1 mAb drastically blocked the upsurge of plasma HMGB1 levels in the SAH rats [37]. This therefore means that, accumulation of blood in the subarachnoid space activated HMGB1 secretion from vascular SMCs in the affected arterial walls Figure 1. This further explains that, the source of the raised plasma HMGB1 is from SMCs in the affected arterial walls [37].

## 5. HMGB1 levels in cerebrospinal fluid during SAH

Several studies have demonstrated that, higher HMGB1 levels in the cerebrospinal fluid (CSF) of SAH patients correlated well with poor outcome [11],[20],[32] Figure 1. Nakahara et al established that, higher HMGB1 levels in CSF was a poor prognostic biomarker in patients with SAH [19]. Also, with a retrospective study utilizing the Hunt and Hess (H&H) grading scale, King et al detected that, the levels of HMGB1 within the CSF tally accurately with neurological outcome [20]. Using the modified Rankin scale, they further indicated that, HMGB1 levels in the CSF highly tally with the magnitude of disability or dependence during follow-up evaluation of patients with SAH. Nevertheless, utilizing the Fisher grade, King et al also establish that the HMGB1 levels in the CSF did not strappingly tally with the manifestation of SAH on computed tomography (CT) scans [20]. Thus, they concluded that HMGB1 was a potential diagnostic biomarker for patients who suffered cerebrovascular accidents (CVA) as well as a prognostic marker for patient outcome after SAH [20].

In affirmative, Bartosz et al with a similar study confirmed that, CSF level of HMGB1 was a novel predictor of outcome following SAH [11] Figure 1. They indicated that expressive levels of HMGB1 as biomarker is as precise as WFNS as well as H&H grading scales. Furthermore, programed monitoring of HMGB1 levels during the entire management period patients with SAH exhibited divergent expressive patterns of HMGB1 which correlated well with the ultimate outcome [11]. They observed that, patients who improved tremendously had constantly low levels of HMGB1 (<10 ng/mL) in CSF during their entire treatment period [11]. Nevertheless, patients with constantly elevated levels of HMGB1 (>10 ng/mL) in the CSF during their entire treatment period had poor prognosis. Survival of patients also correlated well with HMGB1 levels in the CSF. When the HMGB1 levels was lower than 10 ng/mL in two successive samples, the patients survived, but when the levels were constantly above 10 ng/mL, the patients died [11].

## 6. HMGB1 and vasospasm during SAH

Cerebral vasospasm is a delayed and life-threatening clinical complication that arises following SAH [1],[4],[38]. It is considered as anomalous as well as sustained smooth muscle contraction of cerebral arteries. It is established that, following aneurysmal rupture, the cerebral blood vessels in the area of the hemorrhage become persistently constricted [1]. This phenomenon is often linked to delayed neurological deficit as a result of an infarct in the area of brain supplied by arteries involved. The occurrence of vasospasm is often established 3 days following SAH and last for a maximum of 6–8 days [1],[4],[38].

Many substances have been implicated in the evolution of cerebral vasospasm after SAH [4],[38]–[40]. Nevertheless, the multifarious machinery via which the arterial spasm occur is still a matter of debate [1],[32]. It has been demonstrated that, HMGB1 stimulated cerebral vasospasm, augmented gene as well as protein secretory levels of RAGE in neurons following SAH [41]. It is affirmed that, NF-κB is a down-regulator of RAGE. It is established that, the main p65 subunit of NF-κB was expressively higher, signifying that RAGE stimulated the triggering of NF-κB at the immediately after SAH [41]–[43]. Studies have also demonstrated a rise in the mRNA levels for IL-1β, IL-6, IL-8, TNF-α, as well as adhesion molecules during the pathogenesis of aneurysmal SAH triggered vasospasm in rats [44]–[46].

Zhao et al demonstrated that, the artery endothelial cells as well as SMCs in the injured brain area after SAH were triggered resulting in the release of HMGB1, which in turn triggered arterial spasms [41]. Umahara et al detected HMGB1-like immunoreaction in the cytoplasm of vascular SMCs obtained from dead patients with SAH during autopsies studies [47]. Nevertheless, studies have shown that, the degree of angiographic vasospasm does not always correlate with the degree of neurological deficits in SAH patients [32],[48]. Also, plasma HMGB1 level has low accuracy for the prognostication of cerebral vasospasm [32].

## 7. HMGB1 and thrombin levels in SAH

Thrombin is a plasma serine protease, that has been implicates in the configuration of blood clots via cleaving fibrinogen to fibrin [29],[49]. It also has different biological modulatory actions associated with inflammation and thrombosis [29],[50],[51]. It has been established that, hemostatic cascades are activated in a feedback reaction to the vascular wall injury via the extravasated blood in the endothelial extracellular matrix [44],[52]. This phenomenon often results in up-modulation of coagulation cascade sequences such as stimulation of platelets, transformation of fibrinogen to fibrin as well as augmentation of coagulation [44],[53].

Studies have demonstrated that, the lectin-like portion of thrombomodulin binds to HMGB1 in such a way that HMGB1 the thrombin–thrombomodulin complexes can efficiently mortify it to a reduced proinflammatory kind [54]–[56]. It is established that, HMGB1-thrombomodulin complex is able to mollify the proinflammatory properties of HMGB1 without thrombin [54],[55]. Ito et al established a unique anti-inflammatory function of thrombomodulin [54]. They indicated that, thrombomodulin is able to sequester as well as mortify HMGB1, thus averting inflammation caused by HMGB1. It is affirmed that, the lectin-like domain (D1), EGF-like domain as well as the proteoglycan-like domain (D23) of thrombomodulin in a cofactor assay with several thrombomodulin-derived peptides is necessary for the effective cleavage of HMGB1 [54]. Ito et al explained that, D1-bound HMGB1 is cleaved via thrombin connection to nearby thrombomodulin-trans and not to a similar thrombomodulin-cis to which the HMGB1 is concurrently bound [54].

Studies have proven that, HMGB1 and thrombin partakes in major activities resulting in the disruption of the blood brain barrier (BBB) following SAH [18]. HMGB1 and thrombin are both proinflammatory as well as vascular barrier disruptors in many tissues [18],[57],[58]. Therefore, further studies on HMGB1-thrombin complex disruptive abilities following SAH is warranted. Nevertheless, Festoff et al revealed that, thrombin and HMGB1 actively partake in host defense systems by triggering the innate immune system, coagulation as well as inflammation [18]. Haruma et al indicated that, anti-HMGB1 mAb treatment clearly annulled thrombin limit in contractile reaction via protease activated receptor 1(PAR1) stimulation in the SAH rats [37]. They specified that, the regularization of thrombin-triggered contraction of basilar artery was as a result of decrease PAR1 secretion as well as the appropriate hyper-reactive state of SMCs in an ex-vivo experiments using isolated basilar artery [37].

## 8. HMGB1 signaling pathways in SAH

**Figure 2.**
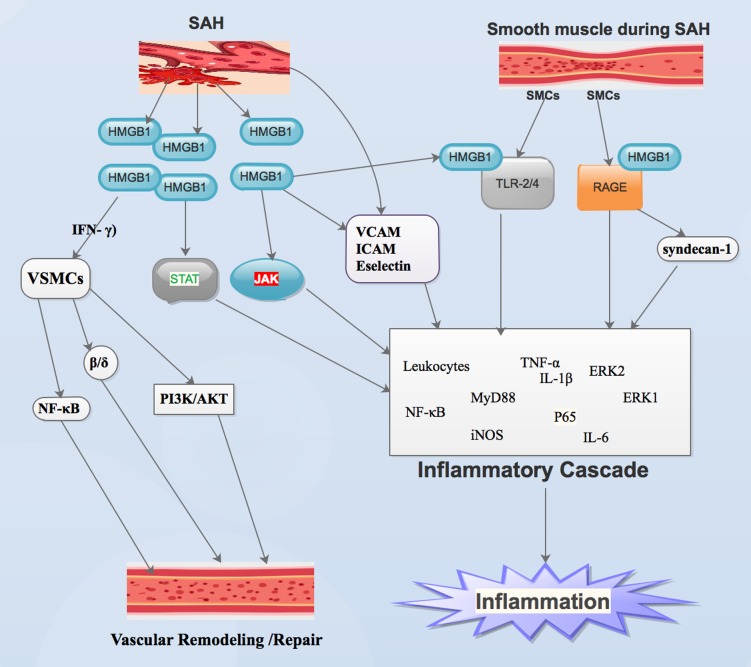
This is an illustration showing the mechanisms via which HMGB1 is able to trigger inflammation as well as Vascular remodeling or repair after SAH.

Studies have shown that debris as well as metabolites from necrotic or stimulated cells triggers inflammation following SAH [59]–[61]. Inflammation usually result in early brain damage following SAH [59],[62]. It is established that the Janus kinase (JAK)-signal transducer and activator of transcription (STAT) pathways intermediate HMGB1 secretion and inflammatory sequels [59],[63]. It is also affirmed that, the JAK-STAT cascade is an essential inflammatory signaling pathway that intermediates immune reactions (Figure 2) [63]. It is extensively secreted by the brain. Its cardinal function is sustenance of equilibrium between pro-inflammation as well as anti-inflammation [59],[60]. An et al demonstrated that, HMGB1 intermediates inflammatory reactions via the JAK-STAT signaling pathway following SAH (Figure 2) [59].

Haruma et al demonstrated that, HMGB1 secreted by SMCs is capable of diffusing to the adjacent cells as well as trigger intracellular reactions via receptor for advance glycation end products (RAGE) and toll-like receptors (TLR2/4) receptors which are secreted by the vascular walls (Figure 2). Several studies have shown that, HMGB1, via binding to RAGE as well as TLR-2 and TLR-4, is capable of stimulating nuclear factor-κB (NF-κB) as well as extracellular regulated kinases (ERK1 and ERK 2) (Figure 2) [44],[64],[65]. It has been established that, TLRs, myeloid differentiation primary response protein 88 (MyD88), NF-κB, interleukin 1β (IL-1β) and tumor necrosis factor-alpha (TNF-α) contributes significantly inflammatory activities following SAH (Figure 2) [23],[39],[66],[67]. It is affirmed that TNF-α intermediates in inflammatory response resulting in the growth of cerebral aneurysm in humans [68],[69]. It is further established that, TNF-α does not only trigger inflammation but also stimulates the inflammatory cascade in response to cerebral aneurysm rupture resulting in SAH (Figure 2) [68],[70].

HMGB1 interacts with receptors signals via diverse pathways. These interactions result in the translocation of NF-κB and P65 to nucleus (Figure 2) [23]. Also, via the same route, stimulation of NF-κB results in the transcription of down-regulatory pro-inflammatory genes such as IL-1β and TNF-α (Figure 2) [23],[71]. It has been demonstrated that, introduction of rHMGB1 into the subarachnoid space resulted in up-modulation of TLR-4 and P65 protein levels as well as down-modulation of inflammatory reactions [71]. This phenomenon affirms that extracellular HMGB1 is capable of stimulating inflammatory reaction as well as the TLR-4–NF-κB signal pathway (Figure 2). This route is one of the many ways HMGB1 triggers inflammatory pathways which in-turn trigger inflammatory cascades [23].

Studies have shown that HMGB1 triggers the secretion of vascular cell-adhesion molecule (VCAM), intercellular adhesion molecule (ICAM), as well as Eselectin. resulting in up-modulation in the conscription of leukocytes (Figure 2) [44],[72],[73]. Haruma et al observed that, anti-HMGB1 mAb absolutely blocked secretion of inflammation-linked genes like IL-6, TNF-α, TLR-4 and inducible nitric oxide synthase (iNOS), in the basilar artery following SAH rats (Figure 2) [37]. It has established that, HMGB1 is capable of binding to heparan sulfate adjacent chains of syndecan-1, a transmembrane proteoglycan [54],[74]. It is also affirmed that, Syndecan-HMGB1-RAGE binding is capable of forming proinflammatory signaling complex (Figure 2) [54],[75]. Therefore, further studies into Syndecan-HMGB1-RAGE pathway during SAH is warranted.

Wang et al. established that HMGB1 is capable of intermediating with interferon-γ (IFN-γ) to trigger phenotypic vascular smooth muscle cells (VSMCs) (Figure 2) [24],[76]. They further stated that base on HMGB1 capacities above, HMGB1 is able to regulate VSMC phenotype switching [24],[76]. Studies have also demonstrated that, HMGB1's ability to regulate VSMC phenotype switching led to enhanced SMCs proliferation as well as migration [76],[77]. Several studies have demonstrated that, stimulation of the Phosphoinositide 3-kinases/protein kinase B (PI3K/AKT) pathway resulted in VSMC proliferation, migration as well as uncontrolled vascular remodeling [24],[78],[79]. Wand et al exhibited that, HMGB1 triggered VSMC phenotypic switching and subsequently vascular remodeling via blockade of the PI3K/AKT pathway during SAH (Figure 2) [24]. Studies have shown that, peroxisome proliferator-activated receptor β/δ pathway as well as the NF-κB pathway participated in vascular remodeling events (Figure 2) [24],[80],[81]. Further studies are warranted to establish whether HMGB1 activated VSMC phenotypic switch via the pathways above.

## 9. Therapeutic potentials of HMGB1 in SAH

**Figure 3.**
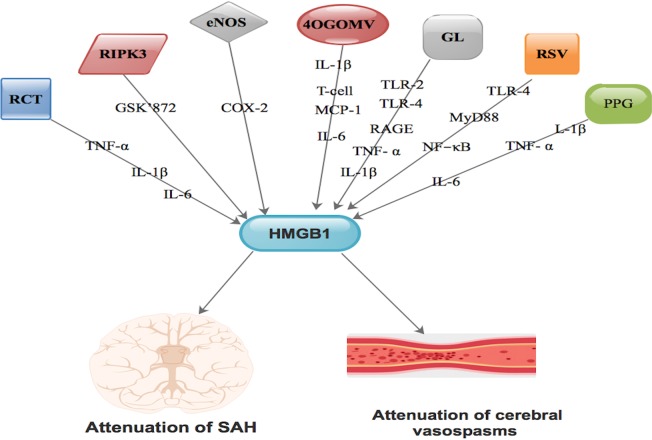
Compounds like resveratrol, glycyrrhizin, rhinacanthin, RIPK3, 4OGOMV, eNOS as well as purpurogallin via different pathways are capable of interacting with HMGB1 resulting in either attenuation of SAH or attenuation of vasospasms or both.

Studies have demonstrated that, HMGB1 has the ability to stimulate neurological recovery as well as blood vessel regeneration through RAGE. Also, HMGB1 has further proven to actively participate at different pathological phases in diverse disease process [42]. It is affirmed that, resveratrol (RSV) function as HMGB1 inhibitor [42],[82],[83]. RSV is derived from dried roots of polygonum cuspidatum [83]. It is also established that resveratrol alleviates pathophysiological processes via HMGB1 blockade [42]. Resveratrol participates actively in SAH-triggered neuronal apoptosis, brain edema as well as nerve damage through blockade of the HMGB1-intermediated TLR-4/MyD88/NF-κB pathway during the initial phase of SAH [42],[82] Figure 3.

Purpurogallin (PPG), a polyphenolic compound is capable of transforming vascular permeability as well as secretion of adhesion molecules, participation in monocyte adhesion and migration via endothelial cells [44],[84]. It is also capable of mitigating SAH triggered IL-1β as well as TNF-α secretion at the early phase of SAH [44]. PPG is capable of averting vasospasm following SAH via inhibiting IL-1β, TNF-α, IL-6 via HMGB1 [44] Figure 3. This compound's ability to downgrade HMGB1 mRNA stimulation led to the activation of microglia interrelated T-cell transmigration as well as IL-6 interrelated delayed inflammatory cascade [44] Figure 3. PPG-HMGB1 pathway is therefore very effective in the prevention of vasospasms during SAH and meritorious of further studies.

Also, 4′-O-β-D-Glucosyl-5-O-Methylvisamminol (4OGOMV), a natural compound, is capable of mitigating SAH by triggering IL-1β as well as MCP-1 secretion at the early phase of SAH [25] Figure 3. It is established that 4OGOMV is able to inhibit proinflammatory cytokines at the initial phase of SAH. It is also able to trigger vascular abnormality as well as attenuate SAH triggered cerebral apoptosis [25]. Furthermore, 4OGOMV is capable of regulating vasoconstriction on rabbit isolated basilar artery rings stimulated by potassium chloride and 5-hydroxytryptamine [25],[85]. Moreover, 4OGOMV is capable of blocking the proliferation of SMCs triggered by TNF-α in a SMC culture [25],[86]. Chang et al. demonstrated involvement of immunosuppressive roles of 4OGOMV on SAH-triggered vasospasm [25]. This indicated that, 4OGOMV is able to down-regulate HMGB1 mRNA stimulation resulting in the activation of microglia-interrelated T-cell transmigration as well as IL-6-interrelated delayed inflammatory cascade leading attenuation of SAH [25] Figure 3.

Rhinacanthin (RCT) is an orthodox preparation mostly used as remedy in numerous diseases [68]. Its active constituents are extracted from the leaves of Rhinacanthus nasutus [68],[69]. It is established that, RCT is capable of stimulating LPS-triggered pro-inflammatory cytokines like IL-6, IL-1β, TNF-α and prostaglandin to initiate secretion of monocytes [69],[87] Figure 3. It affirmed that, RCT is capable of inhibiting HMGB1 mRNA transcription as well as protein secretion in SAH triggered inflammation [68]. It is further established that, during treatment with RCT, HMGB1 facilitated apoptosis as well as vasocontractive effect of vessels during SAH via HMGB1- TNF-α signaling [68] Figure 3.

Studies have demonstrated that, stimulation of receptor-interacting serine/threonine-protein kinase 3 (RIPK3) gene is capable of up-modulating the secretion of HMGB1 in both in vivo and in vitro experiments [88]. It is established that RIPK3-HMGB1 interaction resulted in the translocation of HMGB1 from the nucleus to the cytoplasm leading to boosting of inflammatory reaction [89],[90]. Also, blockade of PIPK3 led to a concurrent decrease in HMGB1 level [89]. Nonetheless, administration of GSK'872 led to a decrease in the level of cytoplasm-HMGB1 positive cells and down-modulated the secretion of HMGB1 [88] Figure 3. Therefore, RIPK3-intermediated necroptosis aggravates neurological function via HMGB1 translocation and consequent inflammation as well as brain edema following SAH [88]. Simultaneous blockade of HMGB1-RIPK3 could be of therapeutic value, thus, further studies should focus on this pathway.

It is established that, down-modulated secretion of the endothelial nitric oxide synthase (eNOS) gene in the basilar artery of SAH rabbits was salvaged using the cyclooxygenase-2 (COX-2) specific blocker [37],[40]. This means that arachidonic acid metabolites contributed to delayed vasospasm following SAH [40]. Nevertheless, down-regulation of nitric oxide generation via eNOS following SAH was facilitated by COX-2 metabolites and not HMGB1 [37] Figure 3. Also, anti-HMGB1 mAb did not trigger SAH-stimulated down-modulation of eNOS secretion [37]. Therefore, amalgamation of anti-HMGB1 and COX-2 blockade is a capable of eliciting synergistic effect on vasoconstriction following SAH. Further studies are needed to establish the therapeutic effect of HMGB1-COX-2 pathway.

It is affirmed that, glycyrrhizin is capable of lessening sensorimotor deficit, BBB permeability as well as down-modulation of mRNA and protein levels of HMGB1 in brain tissue [15]. It has been demonstrated that, the blockade of HMGB1 stimulation by glycyrrhizin considerably down-regulated the secretion of TNF-α and IL-1β [15] Figure 3. These actions above led to assuagement of neuronal cell death as well as apoptosis following SAH [15]. Studies have shown that, interaction between glycyrrhizin and HMGB1 is capable of activating TLR-2, TLR-4 as well as RAGE ligands, which are down-modulators of several cytokines and inflammatory cascades [15],[91]–[93] Figure 3. Glycyrrhizin has demonstrated as a potential therapeutic agent in SAH via HMGB1 [94].

## 10. Conclusions

The expression of HMGB1 from injured cells during SAH is both passive as well as active via translocation from the nucleus to the cytoplasm. Plasma HMGB1 levels are very useful prognosticators of efficient outcome as well as death after SAH. Also, higher HMGB1 levels in the CSF of SAH patients correlated well with poor outcome. Therefore, CSF level of HMGB1 is a novel predictor of outcome following SAH. Nevertheless, the degree of angiographic vasospasm does not always correlate with the degree of neurological deficits in SAH patients. HMGB1 stimulated cerebral vasospasm, augmented gene as well as protein secretory levels of RAGE in neurons following SAH. Therefore, silencing HMGB1 during SAH could be of therapeutic valve. Compounds like resveratrol, glycyrrhizin, rhinacanthin, RIPK3, 4OGOMV, eNOS gene as well as purpurogallin are capable of interacting with HMGB1 resulting in therapeutic benefits following SAH.
